# The Treatment of Gonorrhœa and Gonorrhœal Arthritis

**Published:** 1904-07-23

**Authors:** 


### THE TREATMENT OF GONORRHŒA AND GONORRHŒAL ARTHRITIS.

MR. JONATHAN HUTCHINSON [1] repeats his teaching that the abortive treatment of gonorrhœa ought to be commenced on the first day that the patient comes under observation. He protests against the view that the development of arthritis demands any modification of this doctrine. Such an occurrence is rather a demand for a prompt and early suppression of the discharge, as without question the arthritis is due to absorption either of the gonococcus itself, or of the toxins it produces. Hence the urgency of the claim to get rid as soon as possible of the source of the whole of the mischief. This claim is still further strengthened when it is considered that in occasional instances the consequences of absorption of the gonorrhœal poison are even more serious than the development of arthritis. Thus there have been recorded several instances in which endocarditis [2] has followed gonorrhœa, the circumstances suggesting in the strongest possible manner that the relationship was one of cause and effect. A few examples have also been noted in which gonorrhœa was followed by acute myelitis, and in others there has resulted a generalised pyæmia going on even to a fatal issue. More recently a condition of multiple neuritis [3] has been observed in a case of gonorrhœa. Fortunately these are very exceptional experiences, but their occurrence and their serious character are more than sufficient to emphasise the necessity of removing the cause of such possibilities. Another argument pointing towards the same conclusion is found in the cases, not, it must be admitted, by any means common, in which ophthalmia neonatorum is followed by synovial inflammations. In these instances, almost invariably, the joint conditions rapidly subside and leave no evil consequences behind them, provided, that is, that the ophthalmia is thoroughly and successfully treated. This is a very different picture from that presented by the obstinate and prejudicial character of the gonorrhœal arthritis of adults. The cause of the difference is doubtless to be found in the readiness with which the source of the virus is removed in the one case and the difficulty which attends its removal in the other. The conjunctival sac is easily and completely brought under the influence of remedies which check the suppuration and destroy the gonococcus, whilst the male urethra is a situation much less ready of access. Hence, when joint conditions complicate the existence of gonorrhœa, in the former they are temporary and evanescent, whilst when these conditions take origin in a urethral inflammation they are usually chronic and difficult to remove. Such an experience should lend vigour to the attempt to abort the urethral discharge at the earliest possible moment. The measures advised by Mr. Hutchinson are (1) the injection of a solution of zinc chloride, two grains to the ounce, three or four times a day; (2) free purgation by Epsom salts, which, if steadily continued, prevents any irritating effect from the injection; (3) a half-dram dose of potassium bromide every night at bed time. These methods are to be maintained until all irritation and discharge have ceased and for a week after that event. If signs of synovitis appear the treatment is not to be relaxed, but the patient, in addition, should be ordered full doses of quinine and colchicum. Mr. Jacobson[4] in a lecture on the same subject also insists upon the necessity for a prompt suppression of the urethral discharge. To produce this result he prefers for the first few days the injection of boiled water, afterwards ordinary Watson Cheyne's bougies —one to be introduced and retained for half an hour twice daily. In the case of gonorrhœal arthritis he advocates fixation with gentle pressure to the affected joint and the improvement of the patient's general vigour and vitality. The former result is best secured by plaster of Paris applied when necessary over pads of cotton-wool and followed in due course by a Martin's rubber bandage. Very rarely indeed does gonorrhœal arthritis call for anything in the shape of heroic surgical measures, though occasionally obstinate persistence of synovial fluid may need paracentesis or incision. Generous feeding, together with such tonics as cod-liver oil, strychnine, and the hypophosphites, is essential to meet the bodily and mental depression which almost invariably attend this disease.

[1] Polyclinic, July 1904. [2] Medical Review, vol. ii., p. 160. [3] Lancet, July 9th, 1904. [4] Polyclinic, April 1902.

